# Double-bundle anterior cruciate ligament reconstruction resulted in better International Knee Documentation Committee objective grading at fifteen year follow-up compared to single-bundle reconstruction

**DOI:** 10.1007/s00264-024-06106-7

**Published:** 2024-02-10

**Authors:** Arttu Seppänen, Piia Suomalainen, Tommi Kiekara, Heikki Mäenpää, Heini Huhtala, Timo Järvelä

**Affiliations:** 1 Faculty of Medicine and Health Technology, Tampere University, Arvo Ylpön Katu 34, 33520 Tampere, Finland; 2 Head of Tampere University Hospital Orthopaedics Trauma Unit, Tampere, Finland; 3 Medical Imaging Center, Faculty of Medicine and Health Technology, Tampere University Hospital, Tampere University, Tampere, Finland; 4 Department of Orthopaedics, Faculty of Medicine and Health Technology, Tampere University Hospital, University of Tampere, Tampere, Finland; 5 Faculty of Social Sciences, Tampere University, Tampere, Finland; 6 Sports Medicine and Arthroscopic Center, Hospital Mehiläinen, Tampere, Finland

**Keywords:** ACL, Double-bundle, Single-bundle, Knee

## Abstract

**Purpose:**

The aim of this prospective randomized study was to evaluate whether the use of the anatomic double-bundle (DB) method for anterior cruciate ligament (ACL) reconstruction results in better clinical outcomes and a lower incidence of graft failure compared with the anatomic single-bundle (SB) method. The hypothesis was that DB ACL reconstruction would result in a lower incidence of graft failure.

**Methods:**

Patients were randomly assigned to either the SB group (*n* = 78) or the DB group (*n* = 75). Evaluation included clinical testing, subjective assessments, functional testing, and International Knee Documentation Committee (IKDC) objective grading. Surgical techniques were anatomic, and the rehabilitation protocol was standardized.

**Results:**

At 15-year follow-up, information was available on 100 patients (65%), of whom 55 (36%) were accepted in the final statistical analysis. There were almost three times as many graft failures in the SB group, but the result wasn´t statistically significant. Subjective assessments, knee stability (KT -1000 and pivot shift), range of motion (ROM), and functional one leg hop test showed no statistically significant differences between the groups. However, DB ACL reconstruction resulted in better International Knee Documentation Committee objective grading (*P* < 0.001).

**Conclusion:**

At the 15-year follow-up, double-bundle surgery resulted in significantly better International Knee Documentation Committee objective grading compared to single-bundle surgery.

## Introduction

Anterior cruciate ligament reconstruction is a common surgical procedure to restore knee stability and prevent further injury after ACL tear [1, 2]. The SB technique, which is the most performed procedure and considered the gold standard, uses a single graft to reconstruct the ACL, whereas the DB technique uses two grafts to separately reconstruct the anteromedial and posterolateral bundles of the ACL [3–5]. The debate about which technique is better has been ongoing for many years, with studies reporting contradictory results [6–10]. Our meta-analysis [11] found that DB ACL reconstruction generally results in better restoration of knee laxity and subjective outcomes than SB ACL reconstruction.

Long-term graft failure after ACL reconstruction is estimated to occur in 5–6% of patients [12, 13]. For example, male gender, younger age, a family history of ACL injury, greater tibial slope and return to high activity sports are associated with an increased risk of graft failure [14, 15]. The surgical technique of the ACL also seems to have an influence on the graft failure rate [16, 17].

The purpose of this randomized clinical trial was to evaluate whether the use of the anatomic DB method for ACL reconstruction with hamstring autograft and aperture screw fixation results in better clinical outcomes and a lower incidence of graft rupture compared with the anatomic SB method with hamstring autograft and aperture screw fixation. The study followed patients over a 15-year period and aimed to test this hypothesis.

## Material and methods

### Patients

Ethics Committee of Tampere University Hospital approved the study, and all patients provided written informed consent before participation. Baseline data were collected between 4/2003 and 2/2008 at Hatanpää Hospital, Tampere, Finland. To be eligible for the study, patients had to meet certain inclusion criteria, including a primary ACL reconstruction, closed growth plates, and no ligamentous injuries to the contralateral knee. The inclusion period was 4.8 years and all patients who met the three inclusion criteria were included in the study. In total, 153 patients were randomly assigned to two different groups for ACL reconstruction with hamstring autografts: the anatomic SB technique with interference screw fixation group (*n* = 78) and the anatomic DB technique with interference screw fixation group (*n* = 75). Randomization was performed with closed opaque envelopes. Patients were not blinded to the surgical technique, but were not allowed to reveal the surgical technique to the investigator. All surgical procedures were performed by a single experienced orthopaedic surgeon TJ.

### Evaluation

The evaluation included clinical tests (pivot shift, ROM, anterior tibial translation measured with the arthrometer KT-1000), IKDC objective grading (based on the IKDC knee examination form [18]), subjective assessments (IKDC subjective score, IKDC function score, and Lysholm score), and functional test (one leg hop). The IKDC subjective score is based on the IKDC subjective knee evaluation form [18] and the IKDC function score is the last part of this form. The number of graft failures was assessed by revision surgery. The KT-1000 arthrometer (MEDmetric Corp, San Diego, CA, USA) with a force of 134 N was used. For ROM, the result of the operated leg was compared with that of the non-operated leg. The ROM included lack of passive extension (normal < 3°, nearly normal 3–5°, abnormal 6–10°, and severely abnormal > 10°) and lack of passive flexion (normal 0–5°, nearly normal 6–15°, abnormal 16–25°, and severely abnormal > 25°). One leg hop test was performed to assess the functional capacity of the knee. The patient hopped a maximum length three times on each leg separately and the best result was recorded for both legs. The result of the operated leg was then compared with that of the non-operated leg. The categories were normal (≥ 90%), nearly normal (89–76%), abnormal (75–50%), and severely abnormal (< 50%). All clinical assessments were performed by a blinded and independent investigator AS. Preoperatively, patients' physical demands were rated 1–3 (1 = competitive sport, 2 = recreational sport, 3 = no sport).

### Surgical technique of the double-bundle ACL reconstruction

TJ has previously explained the surgical technique of double-bundle ACL reconstruction in detail elsewhere [19]. Briefly, this procedure first involved comprehensive diagnostic arthroscopic surgery of the knee. This step confirmed the presence of ACL tears and assessed the condition of the meniscus and cartilage. The torn portion of the ACL was then removed, but the tibial attachment site was left intact. No bony notchplasty was performed. Two tunnels were then created on the femoral side through an anteromedial portal (not through the tibia). These tunnels were created manually without a guide to ensure they corresponded to the anatomic position of the insertion site of each bundle. On the tibial side, the tunnels were created using a guide to ensure they matched the anatomic insertion site of the ACL at the tibia. The hamstring grafts (semitendinosus and gracilis) for the procedure were then harvested from the same leg and doubled. These doubled grafts were inserted through the tibial tunnels in a reverse fashion and secured with bioresorbable (specifically D-lactide, L-lactide, and trimethylene carbonate [TMC]) interference screws (Inion Hexalon, Inion Oy, Tampere, Finland). The femoral side was fixed from the inside out, whereas the tibial side was fixed from the outside in.

### Surgical technique of the single-bundle ACL reconstruction

Initially, a diagnostic arthroscopic procedure and debridement were performed as described above. The femoral tunnel was created using an anteromedial portal. A freehand technique was used, and the tunnel was positioned at approximately 10 o’clock in the right knee and at 2 o’clock in the left knee. For the tibial tunnel, a tibial guide was used to ensure it was positioned at the midpoint of the tibial ACL attachment site. The tendons of the semitendinosus and gracilis muscles were then harvested, doubled over, and inserted through the tibial tunnel, extending into the femur, and fixed with metallic (Timoni Company, Kauniainen, Finland) or bioabsorbable interference screws (Inion Hexalon). Both tendons were always harvested and used with both techniques.

### Postoperative rehabilitation

The two groups underwent the same rehabilitation protocol, which included unrestricted range of motion and full weight bearing without a brace. Patients used crutches for three to four weeks and began closed kinetic chain exercises immediately after surgery. They were allowed to begin cycling with an ergometer bicycle after four weeks, running after three months, and pivoting sports after six months postoperatively, provided they had regained full muscle strength and functional stability. If the patient had also undergone meniscal repair during surgery, a range of motion of 0° to 90° was recommended for the first six weeks. Otherwise, rehabilitation was performed according to the methods described above.

### Statistical analysis

Statistical analysis was performed using SPSS version 28.0 (IBM Corporation, Armonk, NY, USA). As all distributions were skewed, the Mann–Whitney U test was used. Frequencies were analyzed with the chi-square test. The significance level was set at *P* < 0.05. Median and IQR were used to describe skewed distributions. To define the minimal clinically important difference (MCID), threshold values of 1 standard error of measurement (SEM), 1.96 SEM, and 2.77 SEM were calculated independently for both groups [20].

## Results

**Fig. 1:**
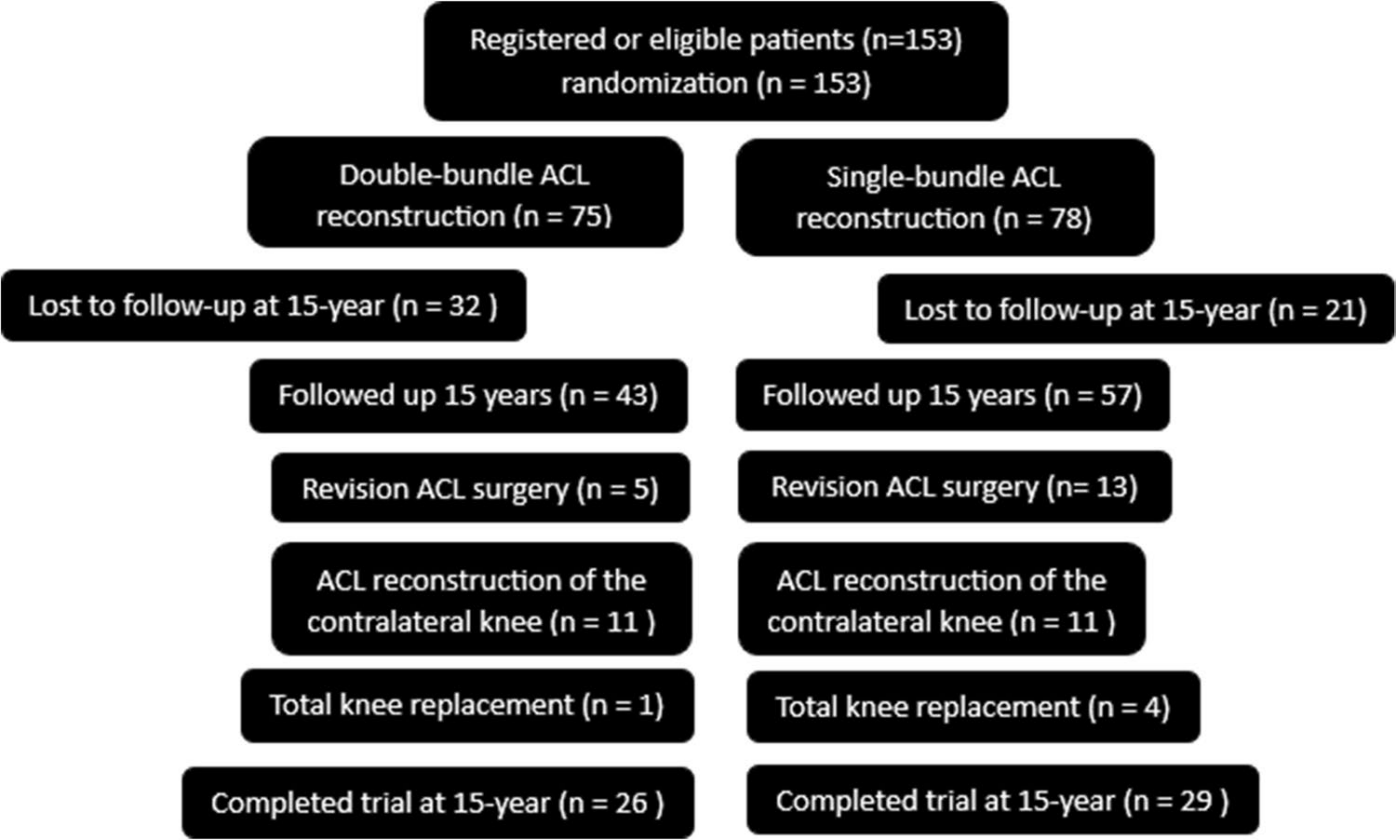
CONSORT (Consolidated Standard of Reporting Trials) flow diagram. *ACL anterior cruciate*

At 15-year follow-up, information on 100 patients (65%), 57 patients from the SB group (73%) and 43 patients from the DB group (57%), was available (Fig. 1). In the SB group, 13 patients (17%) underwent revision surgery because of graft failure, whereas the corresponding number of graft failures in the DB group was five (7%) (n.s.). During the 15-year follow-up period, 11 patients in each group sustained ACL tear in the contralateral knee. Moreover, four patients (5%) in the SB group and one patient (1%) in the DB group underwent total knee replacement (n.s.). Thus, 29 patients from the SB group and 26 patients from the DB group were accepted for statistical analysis. There was no difference in physical demands between the groups (n.s.). Patient demographics are presented in Table 1. The surgical findings of the two study groups are presented in Table 2. The median and mean of the follow-up time were 16.3 years (14.6–17.1) and 15.9 years (1.4), respectively.

**Table 1:** Preoperative demographic data

	DB		SB		
	*n* = 26		*n* = 29		
	n/median	%/Q_1_-Q_3_	n/median	%/Q_1_-Q_3_	*p*-value
Male	16	62	18	62	
Height, cm	175.5	164.75–183	174	164–180.50	n.s
Weight, kg	80	64.75–90.75	85	70–94	n.s
Age, y	36	28–41	36	28.5–42	n.s
BMI	24.8	22.4–28.7	28.4	25.2–30.3	n.s
Physical demand 1–3	5, 16, 4		4, 20,4		n.s

*DB *double-bundle*, SB *single-bundle*, n.s. *not significant

**Table 2:** Meniscal status and treatment at ACL operation

	DB group n (%)	SB group n (%)	*P* Value
Status			n.s
Isolated ACL rupture	7 (27)	10 (34)	
ACL and medial meniscal tear	11(42)	6 (21)	
ACL and lateral meniscal tear	7 (27)	8 (28)	
ACL and both meniscal tears	1 (4)	5 (17)	
Threatment of meniscal tear			
Endoscopic fixation	3	4	
Partial resection	15	13	
Left in situ	1	2	

*ACL *anterior cruciate ligament*, DB *double-bundle*, SB *single-bundle*, n.s. *not significant

The median scores for the IKDC subjective assessment were 85 (78–95) in the DB group and 82 (65–94) in the SB group. Similarly, for the Lysholm score, the median score was 90 (83–97) in the DB group and 89 (76–94) in the SB group. Moreover, the median IKDC function score was eight (7–10) in the DB group and 8 (7–9) in the SB group. No statistically significant differences were found in any of these scores at 15-year follow-up (Table 3).

**Table 3:** Clinical evaluation outcomes

	DB group (*n* = 26)	SB group (*n* = 29)	*P* Value
Lysholm score	Median (IRQ)/n (%)	Median (IQR)/n (%)	
Preoperatively	72 (58–82)	62 (51–72)	n.s
15-year follow-up	90 (83–97)	89 (76–94)	n.s
IKDC subjective score			
15-year follow-up	85 (78–95)	82 (65–94)	n.s
IKDC function score			
Preoperatively	5 (4–5.25)	5 (2.5–5)	n.s
15-year follow-up	8 (7–10)	8 (7–9)	n.s
KT-1000 arthrometer difference, mm			
Preoperatively	5 (3–6)	5 (3–6)	n.s
15-year follow-up	1 (0–2)	1 (-1–2)	n.s
Pivot shift test			
Preoperatively			n.s
Normal	0	0	
Nearly normal	9 (35)	11 (38)	
Abnormal	17 (65)	16 (55)	
Severely abnormal	0	2 (7)	
15-year follow-up			n.s
Normal	23 (88)	23 (79)	
Nearly normal	3 (12)	5 (17)	
Abnormal	0	1 (4)	
Severely abnormal	0	0	
Lack of passive extension			
15-year follow-up			n.s
Normal	18 (69)	21 (75)	
Nearly normal	8 (31)	5 (18)	
Abnormal	0	2 (7)	
Severely abnormal	0	0	
Lack of passive flexion			
15-year follow-up			n.s
Normal	19 (73)	15 (54)	
Nearly normal	6 (23)	11 (39)	
Abnormal	1 (4)	2 (7)	
Severely abnormal	0	0	
IKDC objective score			
Preoperatively			n.s
Normal	0	0	
Nearly normal	0	1 (3)	
Abnormal	25 (96)	23 (79)	
Severely abnormal	1 (4)	5 (17)	
15-year follow-up			< .001*
Normal	12 (46)	1 (3)	
Nearly normal	12 (46)	21 (72)	
Abnormal	2 (8)	7 (24)	
Severely abnormal	0	0	
Functional one leg hop test			
15-year follow-up			n.s
Normal	21 (80)	16 (57)	
Nearly normal	2 (8)	5 (18)	
Abnormal	1 (4)	3 (11)	
Severely abnormal	2 (8)	4 (14)	

*DB* bouble-bundle, *SB* single-bundle, *n.s.* not significant, Lysholm score and IKDC subjective score range: 0–100 (100 = best), *IKDC* function range: 0–10 (10 = best), *Statistically significant result

Anterior tibial translation was measured with the KT -1000 arthrometer. The median difference (index minus opposite) was 1 mm in both groups, and the IQR was -1 to 2 mm in the SB group and 0 to 2 mm in the DB group (n.s.). Knee rotational stability was assessed with the pivot shift test. In total, 23 (79%) patients in the SB group and 23 patients (89%) in the DB group had a negative pivot shift test result (n.s.). However, DB ACL reconstruction resulted in better results in IKDC objective grading (*P* < 0.001). In the DB group, 12 patients (46%) had a normal IKDC objective grading, whereas the corresponding result in the SB group was 1 (3%).

In the DB group, 18 patients (69%) had normal passive extension, whereas 21 (75%) patients had normal passive extension (n.s.) in the SB group. Regarding passive flexion, 19 patients (73%) in the DB group and 15 patients (52%) in the SB group had normal results (n.s.).

One leg hop test was performed to assess the functional capacity of the knee. A total of 21 patients (81%) in the DB group and 16 patients (57%) in the SB group had normal functional results (n.s.). The 15-year follow-up results were significantly better in both groups compared with the preoperative situation in terms of Lysholm score, IKDC function score, IKDC objective grading, and stability measurements (*P* < 0.001).

The percentage of patients achieving the MCID for the Lysholm score was 72% (1 SEM), 72% (1.96 SEM) and 69% (2.77 SEM) in the SB group and 73% (1 SEM), 62% (1.96 SEM) and 58% (2.77 SEM) in the DB group. Correspondingly, the percentage of patients achieving the MCID for the IKDC functions score was 86% (1 SEM), 79% (1.96 SEM) and 79% (2.77 SEM) in the SB group and 81% (1 SEM), 81% (1.96 SEM) and 77% (2.77 SEM) in the DB group.

## Discussion

The main finding of the present 15-year follow-up study was that the double-bundle technique resulted in better IKDC objective grading compared with singe-bundle technique. IKDC objective grading considers the measured variables as a whole. The differences appeared to come primarily from the results of ROM, although there were no statistically significant differences in passive extension or passive flexion. In addition, there were twice as many positive pivot shift test results in the SB group as in the DB group. One possible explanation for this statistically significant result in IKDC objective evaluation could be that the knees in the DB group are more stable and have a better range of motion than the knees in the SB group, although no statistically significant differences were found when the outcomes were analyzed separately. To our knowledge, similar findings comparing anatomic DB reconstruction with anatomic SB reconstruction at mid- or long-term follow-up have not previously been published.

Anterior stability results, measured with the KT-1000 arthrometer, were equal between the groups. In summary, no statistically significant difference was found between the groups in knee stability, including pivot shift test. In their long-term study, Balasingam et al. [21] reported similar results to those in our study. In contrast, Mao et al. [22]found better knee stability results in the DB group compared to the SB group in their long-term retrospective study.

Suomalainen et al. used the same research population as in the present study in their two year follow-up study in 2011, [23] and their main finding was that there were fewer graft failures in the DB group (*n* = 1) than in the SB group (*n* = 7) (*P* = 0.04). Similar results were also obtained in 2012 [24] and 2017 [17] when they had partially the same study population. After 15 years of follow-up, there were nearly three times as many graft failures in the SB group, but the result was no longer statistically significant. This result included previously observed graft failures from previous trials, [17, 23, 24] even if patients had not participated in the 15-year trial. The graft failures in the SB group were all caused by a minor accident. One graft failure in the DB group was due to major trauma with bone fractures, while the others were caused by a minor accident. The tunnel positions of these patients were examined by MRI at the 2-year follow-up and/or at the ACL revision surgery and were all adequate [25, 26]. If only graft failures stemming from minor trauma were considered, a statistically significant difference between the groups emerged. In their RCT study with a five year follow-up, Mohtadi et al. [27] found that graft failure occurred in fewer patients in the patellar tendon SB group than in the hamstring tendon SB and hamstring tendon DB groups.

At two year follow-up [23], seven patients (2 in the DB group and 5 in the SB group) had an invisible graft on MRI assessment. At the 15-year follow-up, two of these patients had undergone total knee replacement, 1 patient (DB) had undergone revision surgery, three patients were accepted for statistical analysis, and one patient (DB) was excluded from statistical analysis because of a contralateral ACL injury. At the two year follow-up, the MRI findings of the invisible grafts appeared to have no clinical significance, but at the 15-year follow-up, one of them had undergone revision surgery and two had undergone total knee replacement, so the invisible graft appeared to have long-term clinical significance in at least some of the patients (3/7).

Subjective assessments of the condition of the knees are one of the most important outcomes when the follow-up period becomes longer. All three subjective assessment forms were better in the DB group, although only slightly. However, a statistically significant difference could not be shown in subjective evaluation. Xu et al. have reported similar results in their new meta-analysis [28] which includes only anatomical ACL reconstructions. However, Eliya et al. found that anatomical DB ACL reconstruction moderately improved Tegner scores over the long term [29].

The major limitation of our study was the relatively low follow-up rate (65%), which can add attrition bias to the study. However, with a follow-up period of 15 years, it is difficult to get patients to participate in the study. Some of the patients became bored with the study, some moved abroad or to a more distant location, and at least one patient died. In addition, the research material was collected during the COVID -19 pandemic, which may have reduced the enthusiasm of patients to participate in the study. Furthermore, because only a few patients were included in the statistical analysis at 15 years, the differences between the groups had to be large for the results to be statistically significant.

The strength of the study was the long follow-up period of 15 years and a study design based on an RCT with a blinded examiner. To date, the follow-up period of our study is the longest on the topic of SB versus DB ACL reconstruction.

Since the ACL DB technique appeared to achieve a better result than the SB technique even at 15-year follow-up, its use in ACL reconstruction is recommended option for conventional SB technique. However, it should be borne in mind that the DB technique is more demanding to perform than the SB technique and is therefore only suitable for experienced ACL surgeons.

## Conclusion

The main finding of this 15-year follow-up study was that the double-bundle technique resulted in better IKDC objective grading. Otherwise, no difference between the groups was demonstrated.

## Data Availability

Only members of the research group have access to the data.

## Code availability

Statistical analysis was performed using SPSS version 28.0 (IBM Corporation, Armonk, NY, USA).

## Abbreviations

*DB*: Double-bundle
*ACL*: Anterior cruciate ligament
*SB*: Single-bundle
*RCT*: Randomized controlled trial
*IKDC*: International knee documentation committee
*ROM*: Range of motion
*SEM*: Standard error of measurement
*MCID*: Minimal clinically important difference
*n.s**.*: Not significant
